# The oxidation and hypoglycaemic effect of sorafenib in streptozotocin-induced diabetic rats

**DOI:** 10.1007/s43440-019-00021-0

**Published:** 2020-01-08

**Authors:** Agnieszka Karbownik, Anna Stachowiak, Hanna Urjasz, Katarzyna Sobańska, Agnieszka Szczecińska, Tomasz Grabowski, Joanna Stanisławiak-Rudowicz, Anna Wolc, Edmund Grześkowiak, Edyta Szałek

**Affiliations:** 1 Department of Clinical Pharmacy and Biopharmacy, Poznań University of Medical Sciences, ul. Św. Marii Magdaleny 14, 61-861 Poznan, Poland; 2 Polpharma Biologics SA, ul. Trzy Lipy 3, 80-172 Gdańsk, Poland; 3 University Hospital of Lord’s Transfiguration, ul. Szamarzewskiego 84/86, Poznan, Poland; 4 Department of Animal Science, Iowa State University, 239E Kildee Hall, Ames, IA 50011 USA; 5 Hy-Line International, 2583 240th Street, Dallas Center, IA 50063 USA

**Keywords:** Sorafenib, Streptozotocin-induced diabetes, Metabolism, Sorafenib-associated hypoglycaemia

## Abstract

**Background:**

Diabetes reduces the activity of CYP3A4 and may increase the exposure for the drugs metabolized by the isoenzyme. Sorafenib is a multi-targeted tyrosine kinase inhibitor (TKI), used for the treatment of advanced renal cell carcinoma, hepatocellular carcinoma and radioactive iodine resistant thyroid carcinoma. The TKI undergoes CYP3A4-dependent oxidative transformation, which may be influenced by hyperglycaemia. The aim of the study was to compare the oxidation for sorafenib between healthy and streptozotocin-induced diabetic rats. Additionally, the effect of sorafenib on glucose levels was investigated.

**Methods:**

The rats were assigned to the groups: streptozotocin-induced diabetic (DG, *n* = 8) or healthy (HG, *n* = 8). The rats received sorafenib orally as a single dose of 100 mg/kg. The plasma concentrations of sorafenib and its metabolite N-oxide were measured with the validated high-performance liquid chromatography with ultraviolet detection.

**Results:**

The difference between groups in *C*_max_ and AUC_0−*t*_ values for sorafenib were significant (*p* = 0.0004, *p* = 0.0104), and similarly for the metabolite (*p* = 0.0008, *p* = 0.0011). Greater exposure for the parent drug and analysed metabolite was achieved in diabetic group. However, the *C*_max_, AUC_0−*t*_, and AUC_0−∞_ ratios between the metabolite and sorafenib were similar in both groups. The significant reduction of glycaemia was observed only in the diabetic animals.

**Conclusion:**

The findings of the study provide evidence that diabetes significantly influence on the exposition for sorafenib and its metabolite, but similar ratios N-oxide/sorafenib for AUC and *C*_max_ in healthy and diabetic animals suggest that oxidation of the TKI is rather unchanged. Additionally, sorafenib-associated hypoglycaemia was confirmed in diabetic animals.

## Introduction

Sorafenib is an anticancer drug belonging to the group of tyrosine kinase inhibitors (TKIs). It restricts tumour cells proliferation and angiogenesis [1]. It inhibits the activity of target enzymes/factors located in tumour cells (CRAF, BRAF, V600E BRAF, c-KIT and FLT-3) and in tumour vasculature (CRAF, VEGFR-2, VEGFR-3 and PDGFR-β) [2]. Since 2005 sorafenib has been used to treat adult patients with advanced renal cell carcinoma (RCC), after failure of first line treatment with tyrosine kinase inhibitors (sunitinib, pazopanib) or cytokine (IFN-α) [3]. Since 2007 sorafenib has been registered to treat patients with hepatocellular carcinoma (HCC) and since 2013—patients with locally advanced or metastatic, thyroid cancer, resistant to treatment with radioactive iodine [4].

Medicals trials shows that diabetes doubles the risk of hepatic, pancreatic and endometrial cancer, and it increases by about 20–50% the risk of colorectal, breast and bladder cancers. Diabetic patients are more likely for cancer diagnosis because of metabolic disorders, which accompany the disease (e.g., hyperglycaemia, insulin resistance, hyperinsulinemia or elevated level of insulin-like growth factor 1). Epidemiological data have shown that diabetes type 2 increases the risk of kidney cancer. It is correlated with its severity, risk of relapse and mortality [5–7]. Obesity patients with diabetes type 2 are more likely to develop HCC. The time of diabetes correlates with the rate of HCC development, and diabetes control can be worse in patients with cirrhosis and HCC [8]. Apart from that, trials have shown that sorafenib is safe to diabetic patients. However, research showed that patients with HCC and diabetes have shorter time progression compared to non-diabetic patients. This may suggest more effective anti-cancer treatment of patients with HCC and diabetes [9]. Diabetes affects the expression of many cytochrome P450 isoforms. The active disease increases the activity of: CYP2E1, CYP 1A1/2, CYP 3A1/2 and decreases the activity of CYP3A4 [10]. It is known that sorafenib is chiefly metabolised in the liver and undergoes CYP3A4-dependent oxidative transformation to sorafenib N-oxide, which possess biological activity similar to the parent drug and represents approximately 9–16% of circulating substances at steady state. Apart from that, sorafenib is a substrate for the P-glycoprotein membrane transporter (P-gp), whose activity also changes in diabetes [11, 12]. P-gp is involved both in the process of drug absorption and excretion. Therefore, a change in the expression of this transporter with concomitant hyperglycaemia increases the risk of changes in sorafenib concentrations in the blood.

The aim of the study was to compare the oxidation for sorafenib between healthy and streptozotocin-induced diabetic rats. Additionally, the effect of sorafenib on glucose levels was investigated.

## Materials and methods

### Reagents

Sorafenib (CAS number 284,461-73-0) and sorafenib N-oxide were purchased from LGC Standards (Łomianki, Poland). Lapatinib used as the internal standard (CAS number 231,277-92-9), streptozotocin (STZ), methanol, acetonitrile, ethyl acetate, glacial acetic acid, ammonium acetate and dimethyl sulfoxide (DMSO) were purchased from Sigma-Aldrich (Poznań, Poland). Citric acid and sodium citrate purchased from POCH (Gliwice, Poland); Nexavar® (sorafenib) was purchased (batch number BXHT61) from Bayer Pharma AG (Warsaw, Poland).

### Animals

The experimental protocol for this study was reviewed and approved by the Local Ethics Committee. All procedures were performed in accordance with European Union regulations concerning the handling and use of laboratory animals. The study was conducted following the principles of the 3Rs (Replacement, Reduction and Refinement). Adult male Wistar-strain rats (weight 400–445 g) were used in the study. The animals were maintained under standard breeding conditions with a 12 h light–12 h dark cycle at constant room temperature (23 ± 2 °C), relative humidity (55 ± 10%) and given *ad libitum* access to feed and water. After acclimatisation the rats were randomly assigned into two groups: the diabetic group (DG) (*n* = 8) and the healthy group (HG) (*n* = 8).

### Induction of model non-insulin dependent diabetes mellitus (NIDDM) by streptozotocin

DG rats were fed a high-fat diet (Labofeed B, 60% fat; Morawski, Poland) for a period of 4 weeks to induce insulin resistance. After 2 weeks of dietary manipulation the animals were intraperitoneally injected with STZ (35 mg/kg body weight in 1000 μL of sterile citrate buffer, pH 4.2). Citrate buffer was injected as a vehicle in HG. The high-fat diet was continued for subsequent 2 weeks in the DG. The rats in the HG were fed with standard diet (Labofeed B, 8% fat) and water *ad libitum*. The rats with the blood glucose level ≥ 250 mg/dL were considered diabetic and qualified for this study. The percentage reduction of the glucose levels in the rats was calculated using the following formula:

$$ \% {\text{Reduction}}_{{{\text{glucose}}}} = \frac{{(V_{0} - V_{{\text{t}}} ) \cdot 100}}{{V_{0} }}, $$

where *V*_0_ glucose concentration at baseline and *V*_t_ glucose concentration at the time of maximum reduction.

After injecting streptozotocin 10% glucose solution was additionally administered for 24 h to prevent hypoglycaemia. Two weeks after streptozotocin had been administered to DB rats and citrate buffer to HG rats, the animals received sorafenib at a dose of 100 mg/kg b.m. The drug was dissolved in 1 mL 10% DMSO and administered orally by means of a gastric feeding tube. 0.1 mL blood samples were collected from rats by cutting a piece of their tails. The samples were collected at the following time points: 0, 0.5, 1, 1.5, 2, 3, 4, 5, 6, 7, 8, 10, 12, 24, 30, 48, 72, 96 h. Each time on blood collection glycaemia was measured.

### HPLC-UV conditions

The concentrations of sorafenib and sorafenib N-oxide were assayed using the high-performance liquid chromatography method with ultraviolet detection (HPLC-UV) [13]. The validation was performed according to the guidelines of the European Medicines Agency (EMA) concerning validation of bioanalytical methods. Chromatographic analysis was conducted on a Alliance® system with 2695 Separation Module and a Dual Lambda (*λ*) absorbance detector (Waters Corporation®, USA). Data acquisition was performed using Empower software v. 1154. Separation was carried out on Symmetry® C8 column (250 mm × 4.6 mm, 5.0 µm particle size, Waters Corporation®, USA). The column temperature was maintained at 25 °C. The mobile phase consisted of ammonium acetate 0.1 M pH = 3.4 (adjusted with glacial acetic acid) (solvent A) and acetonitrile (solvent B). The following gradient was used: the percentage A was decreased from 60 to 29%, while at the same time over 21 min percentage B increased linearly from 40 to 71%. The UV detection wavelength was set at 265 nm, and the injection volume was 20 µL. The volume of the analyzed rat plasma was 25 µL. The samples were determined using liquid–liquid extraction.

### Pharmacokinetic analysis

The pharmacokinetic parameters of sorafenib and sorafenib N-oxide were calculated using a non-compartmental analysis in Phoenix® WinNonlin® 8.1 software (Certara L.P.). The maximum plasma concentration (*C*_max_) and the time to reach the maximum plasma concentration (*t*_max_) were obtained directly from the concentration—time data. The elimination rate constant (*k*_el_) was estimated from the slope of the terminal linear segment of the log mean concentration-time plot using the automatic best-fitting option in WinNonlin 8.1. The elimination half-life (*t*_1/2*k*el_) was calculated from ln2/*k*_el_. The area under the concentration-time curve from zero to the time of the last concentration measured (AUC_0−*t*_) was calculated by a linear trapezoidal rule and the residual area under the curve (AUC_res_) was estimated by extrapolation from the last concentration measured (*C*_last_) to infinity using *C*_last_/*k*_el_ ratio. The area under curve from zero to infinity (AUC_0−∞_) was computed as a sum of AUC_0−*t*_ and AUC_res_.

### Statistical analysis

The traits were tested for departure from normality using the Shapiro–Wilk test. The traits which did not show significant deviation from normality were subject to the heterogeneity of variance test, followed by pooled (heterogeneity of variance test *p *value > 0.05) or Satterthwaite (heterogeneity of variance test *p *value < 0.05) *t* tests to verify the significance of differences between the HG and DG. Differences between the HG and DG in the traits which showed significant departure from normality were tested with the Kruskal–Wallis test. The analysis was performed using capability, *t* test and npar1way procedures of SAS version 9.4. The 90% confidence intervals for the ratio of geometric means were constructed.

## Results

The calibration curve for sorafenib was linear, within the range of 0.025–5.0 µg/mL (*r* = 0.999), for sorafenib N-oxide within the range of 0.02–0.40 µg/mL (*r* = 0.997). The lower limit of quantification (LLOQ) for sorafenib and sorafenib N-oxide were 0.025 and 0.020 µg/mL, respectively. The high precision (coefficient of variation, CV < 10%) and accuracy (%bias ≤ 9%) for sorafenib and sorafenib N-oxide of the applied methodology was obtained.

**Fig. 1:**
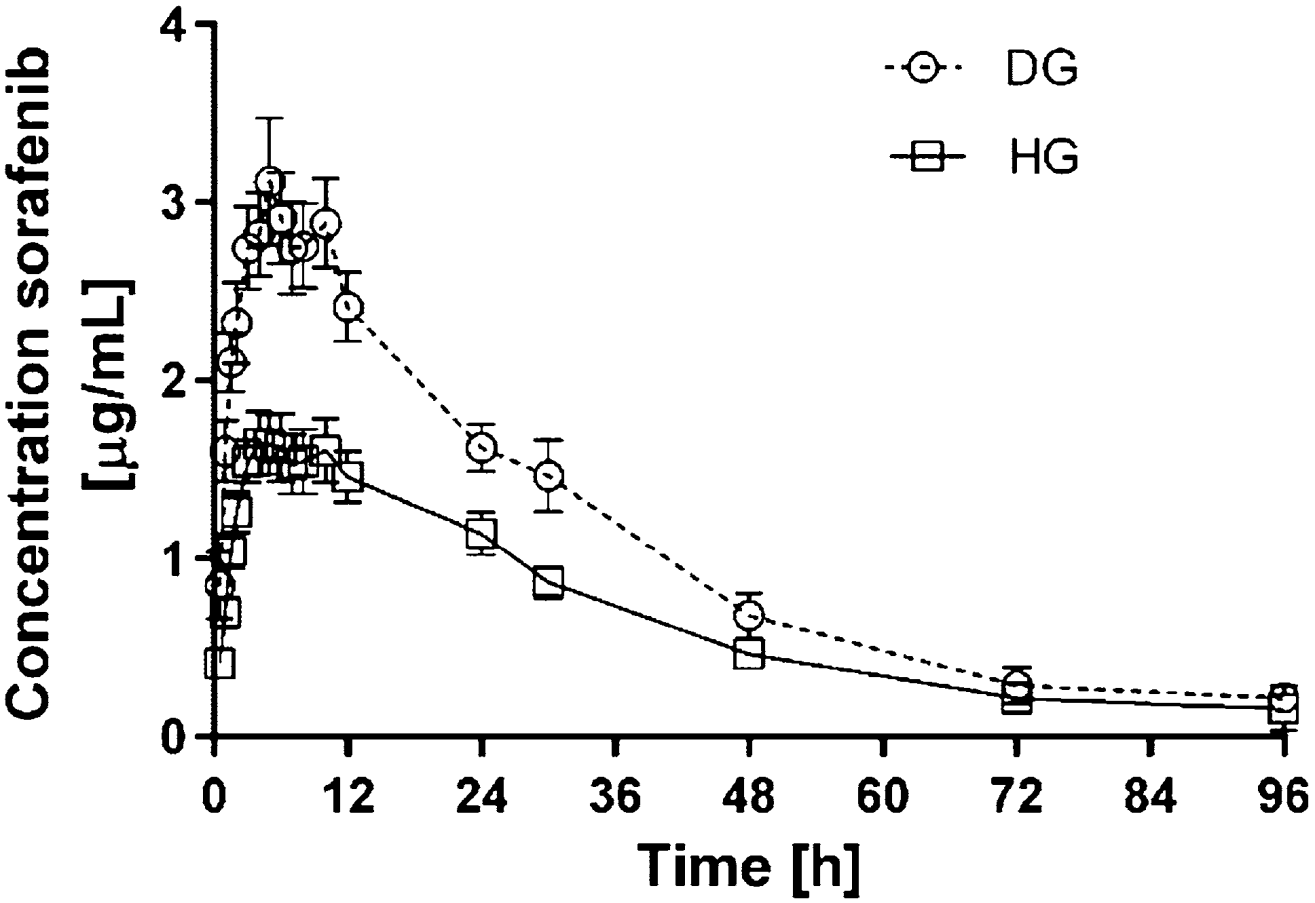
Sorafenib plasma concentration-time profiles following single oral administration of 100 mg/kg of drug in diabetic (DG) and healthy group (HG) of rats (arithmetic means with standard error of mean)

**Fig. 2:**
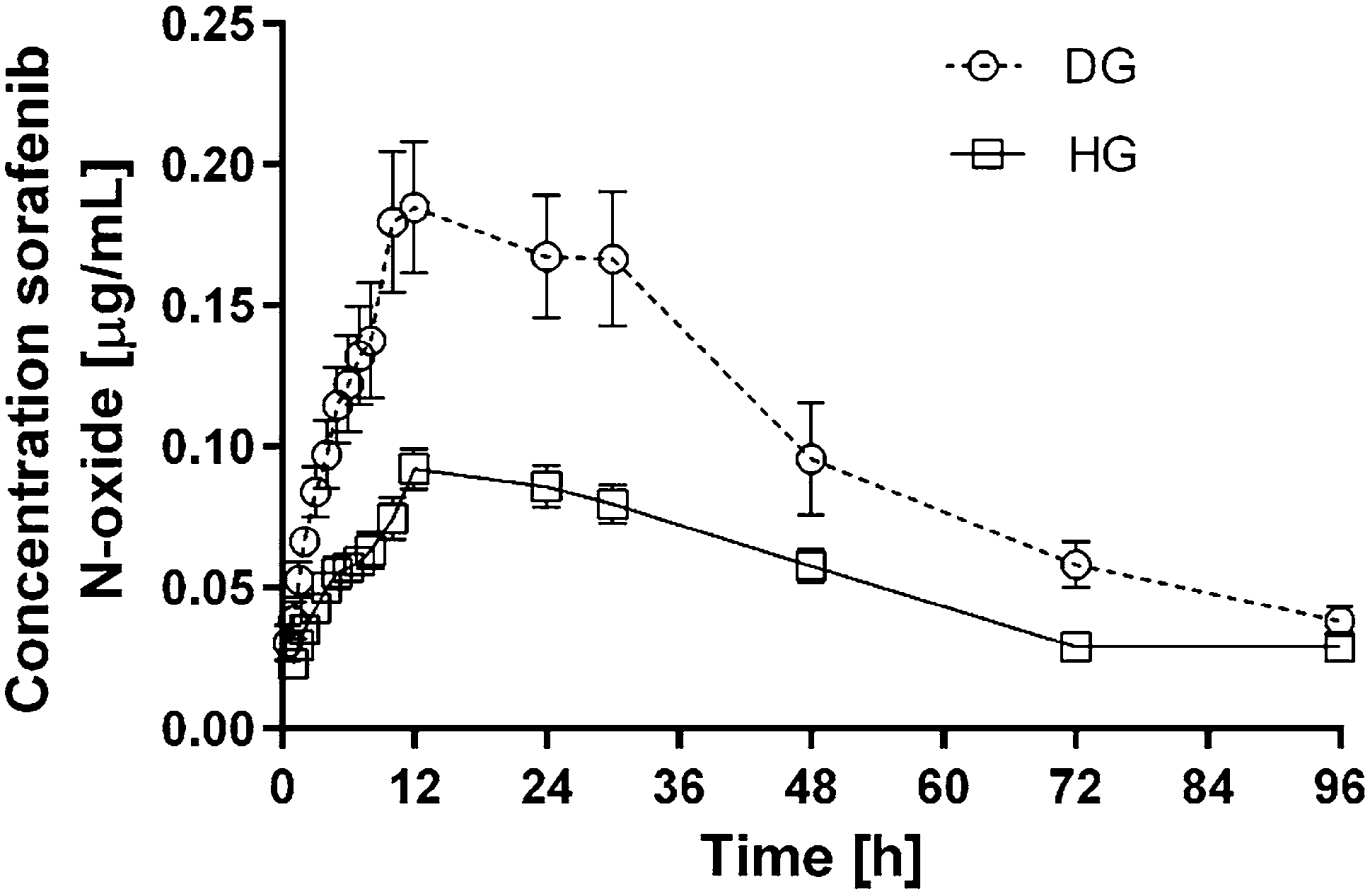
Sorafenib N-oxide plasma concentration-time profiles following single oral administration of 100 mg/kg of drug in diabetic (DG) and healthy group (HG) of rats (arithmetic means with standard error of mean)

All the data were expressed as the mean value ± standard deviation (SD). The groups of rats did not differ significantly in terms of body mass. The arithmetic means of plasma concentrations for sorafenib and its metabolite N-oxide after oral administration to the groups are shown in Figs. 1 and 2. The main pharmacokinetic parameters from non-compartmental methods are summarized in Table 1.

**Table 1:** Plasma pharmacokinetic parameters of sorafenib and its metabolite N-oxide in diabetic group (DG) and healthy group (HG) of rats

Pharmacokinetic parameters^a^	DG (*n* = 8)	HG (*n* = 8)	G_mean_ ratio^b^ (90% CI) DG versus HG
Sorafenib			
AUC_0−*t*_ (µg × h/mL)	100.089 ± 31.204 (31.2)	60.139 ± 22.031 (36.6)	1.69 (1.24; 2.29)
AUC_0−∞_ (µg × h/mL)	105.311 ± 36.532 (34.7)	67.507 ± 31.782 (47.1)	1.61 (1.13; 2.28)
* k*_el_ (1/h)	0.050 ± 0.022 (42.7)	0.043 ± 0.015 (34.7)	1.17 (0.75; 1.80)
* t*_1/2*k*el_ (h)	16.627 ± 7.964 (47.9)	20.386 ± 15.106 (74.1)	0.86 (0.55; 1.33)
* k*_*a*_ (h)	0.693 ± 0.438 (63.1)	0.578 ± 0.221 (38.3)	1.09 (0.74; 1.60)
* C*l/*F* (mL/h)	443.041 ± 148.208 (33.5)	738.976 ± 316.709 (42.9)	0.62 (0.44; 0.88)
* V*_*d*_*/F* (mL)	9327.569 ± 1932.689 (20.7)	17,983.847 ± 6093.406 (33.9)	0.53 (0.42; 0.67)
* C*_max_ (µg/mL)	3.449 ± 0.858 (24.9)	1.828 ± 0.476 (26.0)	1.90 (1.50; 2.45)
* t*_max_ (h)	5.375 ± 2.504 (46.6)	5.000 ± 1.512 (30.2)	1.01 (0.70; 1.46)
MRT_0−*t*_ (h)	24.372 ± 5.753 (23.6)	24.619 ± 5.020 (20.4)	0.98 (0.81; 1.18)
MRT_0-∞_ (h)	28.232 ± 10.037 (35.6)	32.483 ± 16.512 (50.8)	0.89 (0.65; 1.22)
AUMC_0−*t*_ (µg × h^2^/mL)	2580.225 ± 1316.792 (51.0)	1551.911 ± 895.563 (57.7)	1.66 (1.04; 2.64)
AUMC_0−∞_ (µg × h^2^/mL)	3265.249 ± 2157.854 (66.1)	2593.936 ± 2841.241 (109.5)	1.43 (0.75; 2.72)
Sorafenib N-oxide			
AUC_0−*t*_ (µg × h/mL)	9.554 ± 3.909 (40.9)	4.495 ± 1.229 (27.3)	2.06 (1.55; 2.74)
AUC_0−∞_ (µg × h/mL)	11.230 ± 4.198 (37.4)	5.853 ± 1.249 (21.3)	1.85 (1.45; 2.37)
* C*_max_ (µg/mL)	0.202 ± 0.067 (33.2)	0.092 ± 0.020 (22.0)	2.15 (1.68; 2.73)
* t*_max_ (h)	12.750 ± 4.652 (36.5)	13.500 ± 4.243 (31.4)	0.93 (0.74; 1.18)
* k*_el_ (1/h)	0.024 ± 0.005 (19.5)	0.023 ± 0.006 (23.9)	1.04 (0.85; 1.28)
* t*_1/2*k*el_ (h)	29.982 ± 5.572 (18.6)	31.760 ± 8.861 (27.9)	0.96 (0.78; 1.17)
MRT_0−*t*_ (h)	34.749 ± 4.338 (12.5)	31.511 ± 4.470 (14.2)	1.10 (0.98; 1.25)
MRT_0−∞_ (h)	50.112 ± 6.897 (13.8)	52.245 ± 11.772 (22.5)	0.97 (0.83; 1.14)
AUMC_0−*t*_ (µg × h^2^/mL)	338.066 ± 156.832 (46.4)	144.281 ± 58.010 (40.2)	2.28 (1.60; 3.25)
AUMC_0−∞_ (µg × h^2^/mL)	565.209 ± 222.031 (39.3)	304.981 ± 92.686 (30.4)	1.80 (1.33; 2.43)
Ratio sorafenib N-oxide/sorafenib			
AUC_0−*t*_	0.097 ± 0.027 (27.7)	0.078 ± 0.016 (20.4)	1.22 (0.97; 1.54)
AUC_0−∞_	0.111 ± 0.033 (30.0)	0.098 ± 0.031 (31.2)	1.15 (0.85; 1.56)
* C*_max_	0.059 ± 0.015 (24.7)	0.052 ± 0.011 (21.6)	1.13 (0.91; 1.41)

*k*_el_—elimination rate constant; AUC_0−*t*_—area under the plasma concentration-time curve from zero to the time of the last measurable concentration; AUC_0−∞_—area under the plasma concentration-time curve from zero to infinity; *t*_1/2*k*el_—elimination half-life; *C*l/*F*—clearance; *V*_*ss*_/*F*—volume of distribution; *C*_max_—maximum plasma concentration; *t*_max_—time necessary to reach the maximum concentration; MRT_0−*t*_—mean residence time; AUMC_0−*t*_—area under the first moment curve

^a^Arithmetic means ± standard deviations (CV%) are shown with CV (%) in brackets, ^b^ratio of geometric means (*G*_mean_) between groups (%) with the upper and lower bounds of a 90% confidence interval (CI) in the brackets

The results of the study showed substantially increased exposition to sorafenib in the diabetic animals. The AUC_0−*t*_, AUC_0−∞_ and *C*_max_ values of sorafenib were 66.4% (*p* = 0.0104), 56.0% (*p* = 0.0489) and 88.7% (*p* = 0.0004), respectively higher in the DG when compared to the HG. The greater concentration of the drug was reflected by significant decrease of *V*_*ss*_/*F* (*p* = 0.0008) in the DG. The absorption of the drug proved to be comparable in both groups, what was expressed by similar values of k_a_ (*p* = 0.5200) and *t*_max_ (*p* = 0.9134). However, *C*l/*F* of sorafenib showed to be decreased by 40% in diabetic animals. The reduced elimination might have contributed to improved plasma exposition to the drug, but did not significantly influence *t*_0,5_ (*p* = 0.4008)_,_ and MRT_0−*t*_, MRT_0−∞_, AUMC_0−*t*_, AUMC_0−∞_ values (*p* = 0.9292, *p* = 0.5286, *p* = 0.0587, *p* = 0.1722, respectively).

Similarly to the parent drug, the exposition to sorafenib N-oxide has shown to be greater in the DG. It was reflected by significant increase in AUC_0−*t*_, AUC_0−∞_, AUMC_0-t_, AUMC_0-∞_ and *C*_max_ values by 112.55% (*p* = 0.0011), 91.87% (*p* = 0.0011), 134.31% (*p* = 0.0023), 85.33% (*p* = 0.0046), 119.57% (*p* = 0.0008), respectively. Other PK parameters of sorafenib N-oxide did not differ significantly between the groups.

The comparison of exposure ratios of sorafenib and it active metabolite in DG and HG revealed no significant differences. The values of ratios were comparable for AUC_0−*t*_ (*p* = 0.1159), AUC_0−∞_ (*p* = 0.3581) and *C*_max_ (*p* = 0.2073).

**Fig. 3:**
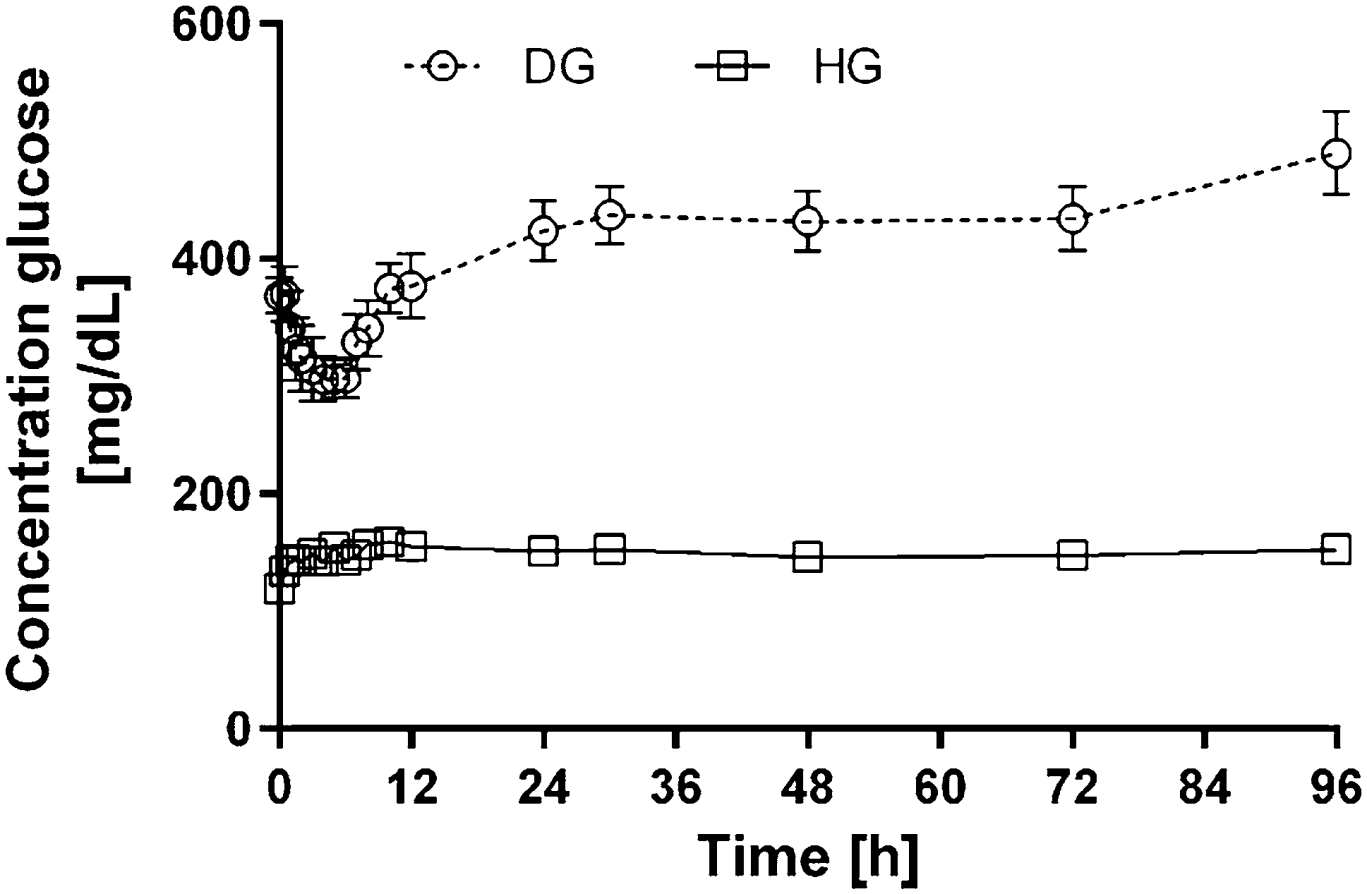
Glucose plasma concentration-time profiles following single oral administration of 100 mg/kg of drug in diabetic (DG) and healthy group (HG) of rats (arithmetic means with standard error of mean)

Figure 3 presents arithmetic means for glucose plasma concentration-time profiles in DG and HG group of rats.

## Discussion

Diabetes and obesity (risk factors for type 2 diabetes) are common comorbidities observed in oncologic patients, which can deteriorate the outcome of various cancers and influence the pharmacokinetics and pharmacodynamics of drugs, including anticancer drugs. Both diseases cause changes in the microbiota, systemic and hepatic inflammation, and they deteriorate the intestinal barrier function. In consequence, intestinal permeability increases [14]. Gastric emptying could be slower in hyperglycaemia state, especially in severe gastroparesis due to autonomic neuropathy observed in diabetes [15]. Diabetes significantly affects the expression of cytochrome P450 isoenzymes. Trials have showed increased CYP2E1 and CYP1A2 activity and reduced expression of CYP3A4 and CYP2C9 [16, 17].

In earlier studies the Authors found significantly increased *C*_max_ and total exposure to sunitinib, erlotinib and lapatinib in diabetic animals, probably in consequence of the inhibition of CYP3A4 and P-gp activity [10, 18, 19]. The PK of sunitinib and lapatinib were analysed in animals with streptozotocin-induced diabetes, whereas the PK of erlotinib was analysed in the rabbits with alloxan-induced diabetes. This study analysed the pharmacokinetics of sorafenib (a new TKI) and its metabolite (N-oxide) in diabetic animals induced by STZ. As CYP3A4 caused intensive metabolism of sorafenib, we could expect similar PK changes to other TKIs. The pharmacokinetics of sorafenib shows high variability (Table 1), but there were significant differences in the following PK parameters: AUC_0−*t*_, AUC_0−∞_, *C*_max_, *V*_*d*_*/F*, and *C*l/*F*. In comparison with the HG group, the *C*_max_ and AUC_0−*t*_ values for sorafenib in the DG group were 56% and 66% higher, respectively. It confirms higher exposure to the drug in DG group and it may suggest that there is real need to adjust sorafenib dosage to diabetic patients. This would be a practical indication helping clinicians to set the dosage regimen of this TKI. Higher TK concentrations may involve a higher risk of adverse effects (AEs). The most frequent AEs of sorafenib correlated with its plasma concentration are: diarrhoea, fatigue, impairment of liver function, anorexia, hand-foot syndrome, alopecia and hypertension [20]. Higher exposure does not seem to be caused by slower metabolism. The absence of statistically significant differences for N-oxide/sorafenib and glucuronide/sorafenib ratios for AUC_0−*t*_, AUC_0−∞_, and *C*_max_ (Table 1) shows that the degree of oxidation of the drug remained rather unchanged. The increase in the concentrations of the metabolites is the consequence of the increased concentration of sorafenib caused by P-gp inhibition [11, 12].

Another aspect of the study was an assessment of the influence of the administration of a single dose of sorafenib on the animals’ blood glucose level. The maximum reduction of glycaemia of 11.2–50.8% was observed in the DG group, and it was observed about 1.5–11 h after the sorafenib administration. The glucose levels did not decrease in the control group. Agostino et al. also observed the hypoglycaemic effect of sorafenib after multiple administration of the TKI to patients [21]. Many other tyrosine kinase inhibitors also exhibited the hypoglycaemic effect in diabetes type 2. The exact mechanism of the TKI-associated hypoglycaemia is still unknown. Suggested mechanisms include the reduction of β-cell apoptosis as well as improvement of peripheral insulin sensitivity through the inhibition of PDGFR and c-Kit [22]. The inhibition of PDGFR increases insulin sensitivity and reduces inflammation in the islets of Langerhans. The inhibition of VEGFR2 or EGFR are believed to be other hypoglycaemic mechanisms in this group of compounds, which reduce inflammation of the pancreas and insulin resistance [23]. According to Prada et al. [24], the hypoglycaemic effect of TKIs is caused by the modulation of macrophage activity, and more specifically—by the transformation of inflammatory macrophages into non-inflammatory forms.

There are some limitations to our study, i.e., the small size of the sample, the potential influence of the diabetogenic drug streptozotocin on the sorafenib PK and the lack of insulin treatment of induced diabetes. Therefore, these are only initial results and the study should be continued to consider additional aspects such as type 1 diabetes, long-lasting diabetes with normo- and hyperglycaemia. Moreover, it will be essential to verify our observations on diabetic patients treated with sorafenib.

In conclusion, this study provides evidence that diabetes significantly influences the pharmacokinetics of sorafenib after single administration, but the similar ratios of N-oxide/sorafenib for AUC and *C*_max_ in the healthy and diabetic animals suggest that the oxidation of the TKI is rather unchanged. Additionally, our research confirmed sorafenib-associated hypoglycaemia in diabetic animals.
