# Correction to: The oxidation and hypoglycaemic effect of sorafenib in streptozotocin-induced diabetic rats

**DOI:** 10.1007/s43440-021-00278-4

**Published:** 2021-06-08

**Authors:** Agnieszka Karbownik, Anna Stachowiak, Hanna Urjasz, Katarzyna Sobańska, Agnieszka Szczecińska, Tomasz Grabowski, Joanna Stanisławiak‑Rudowicz, Anna Wolc, Edmund Grześkowiak, Edyta Szałek

**Affiliations:** 1 Department of Clinical Pharmacy and Biopharmacy, Poznań University of Medical Sciences, ul. Św. Marii Magdaleny 14, 61-861 Poznan, Poland; 2 Polpharma Biologics SA, ul. Trzy Lipy 3, 80-172 Gdańsk, Poland; 3 University Hospital of Lord’s Transfiguration, ul. Szamarzewskiego 84/86, Poznan, Poland; 4 Department of Animal Science, Iowa State University, 239E Kildee Hall, Ames, IA 50011 USA; 5 Hy-Line International, 2583 240th Street, Dallas Center, IA 50063 USA

## Correction to: Pharmacological Reports (2020) 72:254–259 10.1007/s43440-019-00021-0

The article “The oxidation and hypoglycaemic effect of sorafenib in streptozotocin-induced diabetic rats”, written by Agnieszka Karbownik, et al., was originally published electronically on the publisher’s internet portal on 8 January 2020 without open access. With the author’ decision to opt for Open Choice the copyright of the article changed on 8 May 2021 to © Author(s) 2020 and the article is forthwith distributed under a Creative Commons Attribution 4.0 International License, which permits use, sharing, adaptation, distribution and reproduction in any medium or format, as long as you give appropriate credit to the original author(s) and the source, provide a link to the Creative Commons licence, and indicate if changes were made. The images or other third party material in this article are included in the article’s Creative Commons licence, unless indicated otherwise in a credit line to the material. If material is not included in the article’s Creative Commons licence and your intended use is not permitted by statutory regulation or exceeds the permitted use, you will need to obtain permission directly from the copyright holder. To view a copy of this licence, visit http://creativecommons.org/licenses/by/4.0.

The original article has been corrected.

